# 

**Published:** 1950-01

**Authors:** 


### CONTENTS

| | Page |
|---|---|
| **Medicine in Eighteenth-Century Bristol.** By H. J. ORR-EWING, F.R.C.P. | 1 |
| **The XXXVIII Long Fox Memorial Lecture: The Use of Tracer Elements in Biology and Medicine.** By JOHN E. HARRIS. | 10 |
| **Some Post-Mortem Surprises.** By F. D. M. HOCKING, M.SC., M.B., F.R.I.C. | 17 |
| **Some Medical Problems in China To-day.** By CORALIE RENDLE-SHORT, M.B., CH.M., M.R.C.O.G. | 22 |
| **Editorial Notes** | 28 |
| **Obituary** | 29 |
| **Reviews of Books** | 30 |
| **Meetings of Societies** | 34 |
| **Local Medical Notes** | 39 |

